# Supplementary material for: WordCommentsAnalyzer: A windows software tool for qualitative research
Source: F1000Res. 2018 Sep 4;7:536. Originally published 2018 May 3. [Version 2] doi: 10.12688/f1000research.14819.2 (PMC6137411; doi:10.12688/f1000research.14819.2)
Supplement: Supplementary file 3 [file f1000research-7-17614-s0002.tgz › b2a79496-4ada-47c4-9682-a99ce867836d.zip!/Eight Iranian Health Journals/Salmand_Iranian Journal of Ageing _35.docx]

© Salmand_Iranian Journal of Ageing . This work is licensed under CC BY-NC 4.0 (https://creativecommons.org/licenses/by-nc/4.0/).

Volume & Issue: دوره 4 - شماره 4

Title: تأثیر سه نوع درمان ارتزی در کاهش درد در بیماران مبتلا به استئوآرتریت زانو

http://salmandj.uswr.ac.ir/article-1-306-fa.html

اهداف: استئوآرتریت زانو یکی از علل شایع ناتوانی در افراد مسن است. در مطالعه حاضر تاثیر سه نوع ارتز در کاهش دادن درد بیماران مبتلا به استئوآرتریت زانو در ایران ارزیابی شد. مواد و روش‌ها: در این کارآزمایی بالینی آینده نگر سی و شش نفر بیمار مشتمل بر 11 مرد و 25 زن با میانگین سن 54/56±2/82 سال که مبتلا به استئوآرتریت خفیف تا متوسط زانو بودند، شرکت کردند. بیماران در سه گروه دوازده نفره زانوبند نئوپرنی، بریس وزن گیر زانو، و کفی طبی دارای لبه خارجی پاشنه قرار گرفتند و میزان درد آنها با کمک مقیاس خطی–بصری درد، قبل از درمان ارتزی و شش ماه پس از درمان ارتزی با روش آماری تی زوج با یکدیگر مقایسه شدند. یافته‌ها: در هر سه گروه مطالعه، اختلاف معناداری در میزان درد بیماران قبل و بعد از درمان ارتزی دیده شد (P<0/05). درصد کاهش درد در گروهی که از زانوبند نئوپرنی، بریس وزن گیر زانو، و کفی با لبه خارجی استفاده کرده بودند، به‌ترتیب 40/909، 40/186 و 32/493 بود. نتیجه‌گیری: به نظر می رسد که زانوبند نئوپرنی با حفظ گرما در اطراف مفصل زانو باعث کاهش درد بیماران می شود. در گروه بریس وزن گیر زانو، احتمالا بریس با تغییر دادن راستای ران و ساق در زمان راه رفتن باعث تقلیل درد می شود. در گروه کفی طبی با لبه خارجی، تصور می شود که کفی با تغییر دادن وضعیت کالکانئوس به سمت والگوس، باعث کاهش درد شده بود. انجام مطالعات آتی با تعداد شرکت کننده بیشتر و با دوره بازبینی طولانی تر به همراه اندازه گیری میزان جابجایی زانو و کالکانئوس توصیه می شود.

Title: مقایسه کیفیت زندگی سالمندان مقیم خانواده با سالمندان مقیم سراهای سالمندان خصوصی و دولتی شهر تهران

http://salmandj.uswr.ac.ir/article-1-307-fa.html

اهداف: در این پژوهش، کیفیت زندگی و ابعاد تشکیل دهنده آن در سالمندان مقیم خانواده و سراهای خصوصی و دولتی شهر تهران مورد بررسی قرار گرفت. مواد و روش‌ها: مطالعه حاضر در چارچوب مطالعه مقطعی از نوع تحلیلی انجام یافت. برای این منظور تعداد 96 سالمند د سه گروه سالمند مقیم خانواده، سالمند مقیم سرای سالمندان خصوصی و سرای سالمندان دولتی در شهر تهران با توجه به معیارهای پژوهش به عنوان نمونه انتخاب شدند. برای جمع آوری اطلاعات از پرسشنامه مشخصات دموگرافیک و پرسشنامه کیفیت زندگی لیپاد استفاده شد. به منظور تجزیه تحلیل اطلاعات حاصله از آزمونهای کولموگروف–اسمیرنوف، آنالیز واریانس یکطرفه، آزمون تی، آزمون کروسکال والیس و آزمون کای دو استفاده شد. یافته‌ها: نمره کیفیت زندگی سالمندان مقیم خانواده (65/57) بالاتر از کیفیت زندگی سالمندان مقیم سرای سالمندان دولتی (51/30) و خصوصی (50/64) بود. نمره ابعاد عملکرد جسمی، اجتماعی و رضایت از زندگی و نیز مراقبت از خود، سالمندان مقیم خانواده با سالمندان سرای سالمندان اختلاف آماری معناداری نشان دادند (P<0/05). بین کیفیت زندگی سالمندان سرای دولتی و خصوصی و نیز کیفیت زندگی مردان و زنان در واحدهای مورد مطالعه اختلاف معناداری مشاهده نشد. نتیجه‌گیری: سالمندان مقیم خانواده دارای کیفیت زندگی مطلوب تری نسبت به سالمندان مقیم سرای سالمندان خصوصی و دولتی در ابعاد عملکرد جسمی، اجتماعی و رضایت از زندگی بودند.

Title: سطح سرمی منیزیم در زنان یائسه مبتلا به پوکی استخوان

http://salmandj.uswr.ac.ir/article-1-304-fa.html

اهداف: مطالعه حاضر با هدف تعیین وضعیت تغذیه ای و بیوشیمیایی منیزیم در زنان یائسه دچار پوکی استخوان طراحی شد. مواد و روش‌ها: گروه هدف این مطالعه توصیفی – تحلیلی 60 زن یائسه دچار پوکی استخوان مراجعه کننده به کلینیک شیخ الرییس شهر تبریز بودند. دریافت غذایی با استفاده از پرسشنامه یادآمد غذای 3 روزه و بسامد خوراک و اطلاعات فردی و پزشکی افراد با استفاده از دستگاه اسپکتروفتومتری جذب اتمی تعیین شد. آنالیز آماری داده ها با استفاده از روشهای توصیفی و تحلیلی انجام گرفت. یافته‌ها: میانگین سنی زنان 58/85 سال بود. میانگین دریافت روزانه انرژی و پروتئین از رژیم غذایی به ترتیب 1876 کیلو کالری و 53/3 گرم برآورد شد. میانگین دریافت روزانه منیزیم از رژیم غذایی 120 میلی گرم در روز بود که به طور معنی داری پایین تر از مقادیر توصیه شده غذایی (RDA%37) محاسبه شد (P<0/05). میانگین سطح منیزیم سرم در افراد مورد مطالعه 0/7±0/01 میلی مول در لیتر بود که در حد پایین محدوده طبیعی قرار داشت. بین دریافت منیزیم از غذا و سن رابطه معکوس معنی داری (P<0/05) مشاهده شد. نتیجه‌گیری: مطالعه حاضر نشان داد که وضعیت منیزیم در زنان یائسه دچار پوکی استخوان نامطلوب است و با توجه به ارتباط منیزیم با چگالی توده استخوانی و نقش منیزیم بر سلامت استخوان، آموزش تغذیه برای مصرف غذاهای غنی از منیزیم به منظور جلوگیری از کاهش توده استخوانی توصیه می شود.

Title: کیفیت زندگی، رفاه و افسردگی سالمندان ایرانی مقیم سیدنی در کشور استرالیا

http://salmandj.uswr.ac.ir/article-1-301-fa.html

اهداف: هدف از انجام این تحقیق دستیابی به اطلاعات مربوط به وضعیت سلامت سالمندان ایرانی مهاجر در استرالیا بود. مواد و روش‌ها: این بررسی یک مطالعه کمی و به صورت مقطعی بود که در سالهای 2005-7 انجام شد. در این تحقیق تعداد 302 سالمند ایرانی مهاجر که سن آنها 65 سال و بیشتر بود و حداقل شش ماه در سیدنی استرالیا زندگی می کردند، شرکت داده شدند. افراد مورد مطالعه به صورت نمونه گیری تصادفی ساده انتخاب شدند. روش گردآوری داده ها به شکل مصاحبه چهره به چهره، مصاحبه تلفنی و ارسال پرسشنامه از طریق پست و عودت فرم تکمیل شده توسط سالمندان بود. سوالات این تحقیق بر اساس مطالعه وزارت بهداشت و رفاه کشور استرالیا تعیین شد. یافته‌ها: اکثریت سالمندان ایرانی مهاجر دارای علایم و نشانه های اضطراب و افسردگی در حد متوسط بودند (44%) و بیش از نیمی از آنان (58%) سطح متوسطی از احساس روانی خوب بودن را داشتند. از نظر عملکرد فیزیکی (41%) سالمندان دچار محدودیت متوسط و به همان مقدار نیز فاقد محدودیت بودند. از نظر توانایی انجام فعالیت های روزانه زندگی اکثریت (56%) سالمندان مستقل بودند. وضعیت اجتماعی، اقتصادی و فرهنگ پذیری متغیرهای تعیین کننده قوی وضعیت سلامت جسمانی و روانی سالمندان بودند. نتیجه‌گیری: ابتلا به بیماری های مزمن، میزان فعالیت های فیزیکی و اجتماعی و همچنین میزان دسترسی به اطلاعات و آگاهی از خدمات و مراقبتهای سالمندی تعیین کننده های قوی وضعیت سلامت جسمی و روانی سالمندان ایرانی مهاجر بودند.

Title: برخی عوامل مؤثر بر مصرف میوه و سبزی در سالمندان

http://salmandj.uswr.ac.ir/article-1-303-fa.html

اهداف: این مطالعه جهت شناسایی برخی عوامل موثر بر مصرف میوه و سبزی در سالمندان تهرانی انجام گرفت. مواد و روش‌ها: این مطالعه مقطعی بر روی 400 نفر افراد سالمند عضو کانون سالمندی شهر تهران انجام گرفت. از روش نمونه گیری دو مرحله ای جهت انجام نمونه گیری استفاده شد. در مرحله اول، 10 کانون به طور تصادفی از بین کانونهای سالمندی شهر تهران انتخاب و در مرحله دوم برای انتخاب آزمودنی ها از روش تصادفی سیستماتیک استفاده شد. داده های این مطالعه از طریق پرسشنامه ای ساختمند مشتمل بر قسمت های متفاوت (مشخصات دموگرافیک، اطلاعات، منافع و موانع درک شده، مرحله تغییر، خودکارآمدی، میزان روزانه مصرف میوه و سبزی، و حمایت اجتماعی) جمع آوری گردید. از روش های آماری آنالیز واریانس، و آنالیز رگرسیون جهت تجزیه و تحلیل داده ها استفاده شد. یافته‌ها: میانگین مصرف روزانه میوه و سبزی در سالمندان مورد مطالعه، 1/76±1/15، وعده در روز بود که تفاوت معنی داری بین زنان و مردان سالمند از این نظر وجود نداشت (P>0/05) مهم ترین عامل بازدارنده مصرف میوه و سبزی، موانع اقتصادی و مهم ترین عامل تسهیل کنند مصرف، سهولت دسترسی بیان شد. تجزیه و تحلیل رگرسیون نشان داد که با افزایش سطح دانش و منافع درک شده خودکارآمدی و کاهش موانع درک شده، بر میزان مصرف میوه و سبزی افزوده می شود. سالمندانی که در مراحل پیشرفته تر تغییر رفتار از نظر میوه و سبزی قرار دارند از میزان مصرف بالاتر این مواد نیز برخوردارند. نتیجه‌گیری: عوامل فراوانی بر میزان مصرف میوه و سبزی در افراد سالمند موثر است. درک این عوامل به آموزش دهندگان و سایر متخصصان سلامتی در طراحی برنامه های مداخله های مناسب کمک می کند.

Title: افسردگی و عوامل مرتبط با آن در سالمندان اصفهانی (سال 1386)

http://salmandj.uswr.ac.ir/article-1-302-fa.html

اهداف: هدف مطالعه حاضر، شناسایی وضعیت افسردگی در سالمندان و عوامل مرتبط با آن بود. مواد و روش‌ها: این مطالعه، یک مطالعه مقطعی-توصیفی بود. واحد نمونه گیری سالمندان شهر اصفهان در سال 1386 بودند و 248 سالمند (125 مرد و 123 زن) 60 سال به بالا به روش نمونه گیری تصادفی ساده بر اساس خوشه های مربوط به خانوارهای تحت پوشش دانشگاه علوم پزشکی اصفهان که در معاونت بهداشتی این دانشگاه موجود می باشد، انتخاب شدند. وضعیت افسردگی در آنها با مقیاس افسردگی سالمندان که برای سالمندان ایران استاندارد شده، مورد ارزیابی قرار گرفت. جهت سنجش ارتباط بین افسردگی و فاکتورهای دموگرافیک از مدل تحلیل تفکیکی استفاده شد. یافته‌ها: از 125 سالمند مرد و 123 سالمند زن، 121 (96/8%) سالمند مرد و 110 (89/4%) سالمند در گروه سنی 74-60 سال و 4 (3/2%) سالمند مرد و 13 (10/6%) سالمند زن در گروه سنی 75 سال و بالاتر قرار داشتند. %36/6 سالمندان فاقد افسردگی و 63/7% مبتلا به افسردگی بودند که %40/7 افسردگی متوسط 23% افسردگی شدید داشتند. سطح تحصیلات دیپلم و پایین تر از آن (CI0/95: 0/2-0/85 و OR=2/14) عامل خطر برای ابتلا به افسردگی متوسط بود اما جنسیت مؤنث (CI0/95: 0/2-0/85 و OR=0/42) و داشتن همسر در قید حیات و زندگی مشترک با وی (CI0/95: 0/19-1/001 و OR=0/44) نقش حفاظتی در برابر ابتلا به افسردگی متوسط داشتند. داشتن همسر در قید حیات و زندگی مشترک با وی (CI0/95: 0.14-0/85 و OR=0/34) نیز، نقش حفاظتی در برابر ابتلا به افسردگی شدید داشت. نتیجه‌گیری: با توجه به شیوع بالای افسردگی در سالمندان و نقش مهم زندگی تنها در ایجاد آن، به کارگیری مداخلات لازم از جمله آموزش علایم ابتلا به افسردگی جهت تشخیص زودهنگام و مراجعه به موقع سالمند جهت درمان بیماری، بسترسازی فرهنگی و ایجاد زمینه های لازم برای ازدواج مجدد سالمندان تنها، ضروری می باشد.

Title: عوامل مؤثر بر فعالیت فیزیکی در سالمندان منطقه 17 تهران

http://salmandj.uswr.ac.ir/article-1-305-fa.html

اهداف: هدف از این مطالعه تعیین عوامل و موانع فعالیت فیزیکی سالمندان بود. مواد و روش‌ها: این مطالعه به صورت مقطعی در 153 نفر از سالمندان منطقه 17 تهران با روش نمونه گیری تصادفی طبقه بندی شده انجام شد. بدین منظور پرسشنامه محقق ساخته پس از فراوری به صورت چهره به چهره توسط پرسشگران تکمیل شد. جهت تحلیل نتایج از آمار توصیفی و آزمون غیر پارامتری من ویتنی استفاده شد. یافته‌ها: عوامل اصلی مشوق فعالیت فیزیکی سالمندان عبارت بودند از: حفظ استقلال و عدم وابستگی، احساس کارآمد بودن، جلوگیری از خستگی، خرید مایحتاج زندگی، احساس توانایی، نیاز سن بالا به ورزش که همگی جزء عوامل مرتبط با سلامت هستند. اصلی ترین عوامل بازدارنده فعالیت فیزیکی بیماری های مزمن و ناتوانی یا درد بود. مقایسه دو جنس نشان داد که عوامل تفریحی (P<0/01)، عوامل فیزیکی محیط (P<0/01) و عوامل اجتماعی (P<0/05) در مردان بیش از زنان مشوق فعالیت فیزیکی بود. زنان نیز بیش از مردان با موانع مربوط به سلامت (P<0/01) و محیط اجتماعی (P<0/05) مواجه هستند. نتیجه‌گیری: بر اساس یافته ها برای ترویج فعالیت فیزیکی سالمندان بهترین راه معرفی اثرات فعالیت فیزیکی بر سلامت جسمی، روانی و اجتماعی آنان است. برای برطرف سازی موانع نیز مهم ترین کار ارایه برنامه ورزشی مختص سالمندان زیر نظر متخصین است.

Title: تاثیر مشاوره گروهی شناختی بر میزان اضطراب و افسردگی سالمندان زن مقیم آسایشگاه در شهرستان سبزوار در سال 1386- 1387

http://salmandj.uswr.ac.ir/article-1-300-fa.html

اهداف: هدف اصلی این پژوهش تعیین تاثیر مشاوره گروهی شناختی بر میزان اضطراب و افسردگی سالمندان زن مقیم آسایشگاه سالمندان است. مواد و روش‌ها: طرح این پژوهش آزمایشی با پیش آزمون و پس آزمون و گروه کنترل بود. جامعه آماری در این تحقیق کلیه سالمندان ساکن آسایشگاه مادر در شهرستان سبزوار بودند. روش نمونه گیری در این تحقیق تصادفی ساده بود. ابتدا پرسشنامه سلامت عمومی اجرا شد و کسانی که در عامل های اضطراب و افسردگی نمره بالاتر از 6 کسب کردند شناسایی شده و تعداد 30 نفر از آنها به طور تصادفی انتخاب شدند. سپس این تعداد در دو گروه 15 نفری (گروه آزمایش و گروه گواه) به طور تصادفی جایگزین شدند. یافته‌ها: تحلیل نتایج با استفاده از آزمون آماری تحلیل کواریانس انجام شد که نشان داد مشاوره گروهی شناختی به طور معناداری منجر به کاهش افسردگی و کاهش اضطراب شد. نتیجه‌گیری: روش مشاوره گروهی شناختی در کاهش اضطراب و افسردگی تاثیر مثبت دارد و با برگزاری کارگاه های آموزشی برای کارکنان و پرستاران مراکز نگهداری سالمندان می توان از این روش به عنوان درمان کمکی در کنار درمان دارویی استفاده کرد.
